# A Transfer Learning Approach on the Optimization of Edge Detectors for Medical Images Using Particle Swarm Optimization

**DOI:** 10.3390/e23040414

**Published:** 2021-03-31

**Authors:** Delia Dumitru, Laura Dioșan, Anca Andreica, Zoltán Bálint

**Affiliations:** 1 IMOGEN Research Institute, County Clinical Emergency Hospital, 400006 Cluj-Napoca, Romania; lauras@cs.ubbcluj.ro (L.D.); anca@cs.ubbcluj.ro (A.A.); zoltan.balint@phys.ubbcluj.ro (Z.B.); 2 Faculty of Mathematics and Computer Science, Babeș–Bolyai University, 400084 Cluj-Napoca, Romania; 3 Faculty of Physics, Babeș–Bolyai University, 400084 Cluj-Napoca, Romania

**Keywords:** edge detection, evolutionary algorithms, cellular automata, particle swarm optimization, image processing, transfer learning, cardiac MRI

## Abstract

Edge detection is a fundamental image analysis task, as it provides insight on the content of an image. There are weaknesses in some of the edge detectors developed until now, such as disconnected edges, the impossibility to detect branching edges, or the need for a ground truth that is not always accessible. Therefore, a specialized detector that is optimized for the image particularities can help improve edge detection performance. In this paper, we apply transfer learning to optimize cellular automata (CA) rules for edge detection using particle swarm optimization (PSO). Cellular automata provide fast computation, while rule optimization provides adaptability to the properties of the target images. We use transfer learning from synthetic to medical images because expert-annotated medical data is typically difficult to obtain. We show that our method is tunable for medical images with different properties, and we show that, for more difficult edge detection tasks, batch optimization can be used to boost the quality of the edges. Our method is suitable for the identification of structures, such as cardiac cavities on medical images, and could be used as a component of an automatic radiology decision support tool.

## 1. Introduction

Edge detection is an important tool in many computer vision tasks. To solve the problem of edge detection, the first algorithms relied on local pixel information without prior knowledge about the image. Subsequently, newer edge detectors (e.g., based on neural networks) learn abstract representations of the data from an *a priori* training process [1].

We use cellular automata (CA) for this task because cellular automata are discrete local models that are easily adaptable for computer vision problems [2]. They are intrinsically parallel models, which facilitates an efficient implementation, and they operate on local neighborhoods, thus they work well for measuring local disparities in pixel values [3,4].

The edge detection problem has been approached using cellular automata with fixed rules, e.g., linear rules [5] or custom, threshold-based rules [6]. Cellular automata models have also been applied to image segmentation, a related computer vision problem [7]. Additionally, there are methods which rely on automatically finding suitable rules by performing an exhaustive search [8], or by applying other models for this task, such as cellular learning automata [9] or particle swarm optimization (PSO) [10]. In Reference [10], the evolutionary model optimizes a two-step fuzzy cellular automaton rule. There are other approaches based on fuzzy logic, such as Reference [11], which uses cuckoo search and genetic algorithms to optimize fuzzy rules for edge detection, or Reference [12], which proposes framework for the dynamic adaptation of PSO parameters.

As an optimizer for the cellular automata rule, we use particle swarm optimization (PSO), a population-based optimization model. One of the advantages of using this algorithm is that the optimization problem does not need to be differentiable, as it is the case with gradient-based methods [13]. The disadvantage of not being guaranteed to find an optimal solution is mitigated by the swarm memory feature, which prevents the degradation of partial solutions and makes this method perform well in local search problems [14,15]. Swarm memory also helps the model converge faster and thus require a smaller computational cost compared to other evolutionary algorithms [13].

In our approach we combine the use of local information and the adaptive component by optimizing the parameters of a cellular automata rule using PSO. In our previous research, we introduced an optimization framework for edge detectors based on transfer learning [16]. We used a synthetic set of images to optimize the cellular automata rule, which we then applied on a test set of cardiac magnetic resonance imaging (MRI) scans. In Reference [17], we improved our edge detection framework and outperformed the Canny edge detector on a subset of the Brodatz dataset [18]. In this paper, we apply our refined model for the problem of edge detection for cardiac MRI, with the goal of identifying the boundaries of the cardiac cavities.

### Original Contribution

Our main contribution regards the adaptability of the edge detector, given by the PSO optimization step and enhanced by a transfer learning technique.

First, we prove the adaptability of our edge detection framework. For this purpose, we use a test set of MRI scans from our in-house performed clinical study (*Imaging-based, Non-invasive Diagnosis of Persistent Atrial Fibrillation—imATFIB*). This test set contains examples with various properties in terms of grey levels and image noise. We found that, instead of applying the same rule to the entire set, we can split it into two categories based on these disparities, optimize separate CA rules for each, and improve the quality of the edges.

Furthermore, to increase the power of generalization within these two datasets, we explored the idea of optimization on image batches and found that, in certain cases, a well-chosen batch size can further improve the resulted edge detector. Additionally, we used the transfer learning technique from machine learning, which consists of training a model on a set of examples and applying it on a different set of related examples [19]. In our case, we optimized the CA rule on synthetic images created to emulate the properties of the MRI test set, thus minimizing the need for manually annotating medical data, which can be time-consuming and requires expert knowledge [20].

## 2. Materials and Methods

### 2.1. Edge Detection

**Figure 1:**
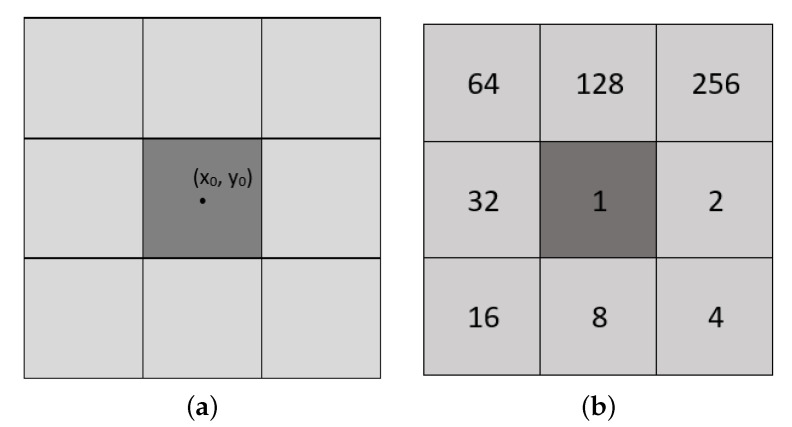
(**a**) A visual representation of the Moore neighborhood with radius 1. (**b**) A visual representation of the nine fundamental rules for a 2D cellular automaton with a Moore neighborhood.

We define edge detection as the problem of identifying sharp disparities in pixel values within a local neighborhood of an image. The instrument that outputs a binary edge map from a grayscale image received as input is called an edge detector. In our approach, we work with the Moore neighborhood, which defines the neighbors of a given point $\left(x_{0},y_{0}\right)$ in a two-dimensional image as

(1) $$N_{\left(x_{0},y_{0}\right)}=\{\left(x,y\right):|x-x_{0}|\leq r,|y-y_{0}\left|\leq r\right\},$$

where *r* represents the radius of the neighborhood, which we set to 1 [21,22]. A visual representation of the Moore neighborhood can be seen in Figure 1a.

Edge detectors can be split in two main categories: the ones that use local information to label edges and no a priori knowledge about the images (such as Sobel, Prewitt, or Canny), and contextual detectors which make use of a priori knowledge [22].

The Canny edge detector [23] is based on three edge detection criteria: good detection or maximization of the signal-to-noise ratio, good localization of the edges, and a single response to an edge. The Canny edge detector consists of smoothing the input image using a Gaussian filter, followed by considering the points located at the maxima of the gradient modulus in the gradient direction. The selected points are then categorized based on a double thresholding step: if the gradient is greater than the higher threshold, the point is labeled as a *strong edge*, if it falls between the two threshold values, it is labeled as a *weak edge*; otherwise, it is discarded. The last step is selecting only the strong edges and the weak edges that are connected to a strong edge [22,23].

### 2.2. Cellular Automata

A cellular automaton is defined by the five-tuple $CA=\{C,N,S,s_{0},\rho \}$, where *C* represents a set of cells, *N* with |N|=n their neighborhoods, and *S* is a set of possible states which are assigned with a transition rule $\rho :S^{n}\to S$, starting from an initial state $s_{0}\in S$ [24].

In this paper, we use linear transition rules, which are obtained through EX-OR operations among the neighbors [4]. We represent a linear rule by a binary number in the following way: we assign the value 1 to a neighbor that we take into account when computing the next state and the value 0 to a neighbor that we do not take into account. In Figure 1b we define a convention by which we assign powers of 2 to the neighbors. Using this convention in conjunction with the binary representation, we can map the neighbors that contribute to the next state to a binary number, which we use to identify the linear rule [2,3,25].

### 2.3. Cellular Automaton Model

We use the representation of Reference [10] in which a pixel at position (i,j)—denoted $X_{i,j}$—in the input image corresponds to a cell of the automaton. The first step of the transition rule is computing the edge membership value according to a linear rule:

(2) $$\mu \left(X_{i,j}\right)=\frac{\phi (X_{i,j})}{\Delta +\phi (X_{i,j})},$$

where $\phi \left(X_{i,j}\right)=\sum_{k}\sum_{l}\left|X_{i,j}-X_{i+k,j+l}\right|$, Δ∈{0,…,255}, and *k*, *l* are selected from {−1,0,1}.

The second step of the transition rule is passing the obtained values through a threshold function F:X↦{0,1} given by:

(3) $$F\left(X_{i,j}\right)=\{\begin{array}{cc} 1, & \mathrm{if}\;\mu (X_{i,j})>\tau \\ 0, & \mathrm{if}\;\mu (X_{i,j})\leq \tau , \end{array}$$

where τ∈[0,1).

In this composite rule there are three parameters that require optimization: Δ, which is inversely proportional to the number of detected edges, the linear rule, which controls the neighbors that we consider when computing the edge membership, and τ, which controls how many points pass as edges.

### 2.4. Particle Swarm Optimization

Particle Swarm Optimization (PSO) is a population-based optimization model which improves the candidate solutions, known as particles, iteratively with respect to a measure of quality or *fitness function* [14].

We define a particle *k* by its *position* $x_{k}$ and its *velocity* $v_{k}$. The swarm moves across the search space at each time step *t* and every particle changes its position based on the velocity, defined as:

(4) $$v_{k}\left(t+1\right)=w*v_{k}\left(t\right)+r_{1}*c_{1}*\left(p_{best_{k}}-x_{k}\left(t\right)\right)+r_{2}*c_{2}*\left(g_{best}-x_{k}\left(t\right)\right),$$

where *w* controls the oscillation of the particle, $p_{best_{k}}$ is the personal best position of the particle *k*, $g_{best}$ is the global best position in the swarm, $c_{1}$ and $c_{2}$ are the swarm history and swarm influence factors, respectively, and $r_{1},r_{2}\in \left(0,1\right)$ are random uniform variables. $x_{k}\left(t\right)$ is updated by

(5) $$x_{k}\left(t+1\right)=v_{k}\left(t+1\right)+x_{k}\left(t\right),$$

and it represents the particle position at time *t* [13,14].

### 2.5. PSO Optimizer

We use PSO to optimize the Δ, τ and linear rule *r* from the cellular automaton rule described in Section 2.3. A candidate solution is represented by the triplet (Δ,τ,r), where Δ and τ are defined in Equations (2) and (3), and r∈{0,…,511}. We normalize this representation by mapping Δ and *r* to a continuous domain and we obtain the final representation $\left(\Delta^{\prime },\;\tau ,r^{\prime }\right)$, where $\Delta^{\prime }=\Delta /255$ and r=r/511.

We use the *Dice Similarity Coefficient (DSC)* [26] to measure the fitness of the particles, which is defined as

(6) DSC=(2·TP)/(2·TP+FP+FN),

where TP,FP, and FN represent the number of true positives, false positives, and false negatives, respectively. In the edge detection problem, we consider the positive class to be an edge point and the negative class a non-edge point. The fitness function is the DSC value for the edge map obtained by applying the optimized CA rule. We choose this fitness function because it accounts for the class imbalance in an edge image (the points corresponding to the negative class outnumber the points corresponding to the positive class), as opposed to alternative metrics, e.g., accuracy.

We optimize the edge detection rule on a set of images to achieve generalization and to eliminate the overhead of repeating the optimization step for each input image.

## 3. Results

### 3.1. Experimental Setup

#### 3.1.1. Edge Detection Framework

We validate our experiments by comparing the results with the Canny edge detector implemented in the Scikit-image library [27]. To use a similar framework to the Canny edge detector, we test our method under three scenarios:

1. applying the CA rule with no additional processing—CA−ED;
2. applying the CA rule followed by a post-processing step—$CA-ED_{post}$;
3. pre-processing the input, followed by applying the CA rule and then the post-processing step—$CA-ED_{pre-post}$.

For post-processing, we introduce thinning and the removal of disconnected edges, similarly to the Canny method. For pre-processing, we apply a Gaussian filter on the input images as the Canny edge detector does, and we test multiple standard deviations for this filter, which we denote by $\sigma_{smooth}$ [23].

#### 3.1.2. Optimizer Setup

We parameterize the PSO optimizer as follows: we use a swarm of 100 particles, we iterate the algorithm for 25 epochs per input image, and we choose ω=0.05, $c_{1}=2.1$, $c_{2}=1.2$. For the Canny edge detector, we use the default threshold values in the Scikit-image library, specifically 10% and 20% of the maximum value of the data type (255 in our case), respectively.

#### 3.1.3. Metrics

We use two metrics to quantify the edge quality: *Peak signal-to-noise-ratio* (PSNR) [28], which indicates the amount of noise in an image with respect to the amount of information, and *Structural Similarity* (SSIM) [29], which evaluates the perceptual similarity between the result and the ground truth.

#### 3.1.4. Dataset

The optimizer is tested on a subset of the cardiac MRI data taken from our in-house performed clinical study—Imaging-based, Non-invasive Diagnosis of Persistent Atrial Fibrillation (imATFIB). The study is registered at clinicaltrials.gov (NCT03584126) and obtained ethical approval from the local Ethics committee (Nr. 20117/04.10.2016). All subjects gave their written informed consent to participate in the study. Patients and healthy volunteers underwent cardiological evaluation using ECG and echocardiography, followed by cardiac MRI measurements with a 3T whole-body MRI system (3.0T Discovery MR750w General Electric MRI scanner) using a dedicated body coil for signal reception.

**Figure 2:**
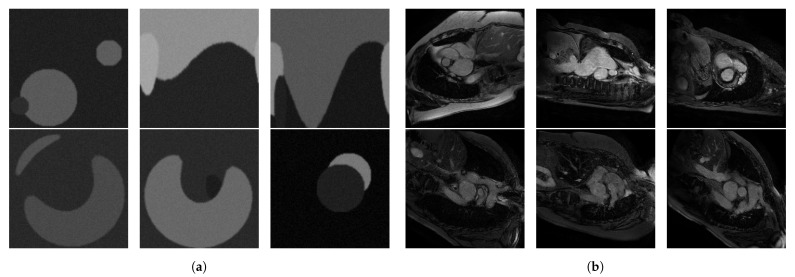
(**a**) Low intensity optimization set, and (**b**) 6 representative images from the low intensity test set (Imaging-based, Non-invasive Diagnosis of Persistent Atrial Fibrillation (imATFIB) clinical study).

**Figure 3:**
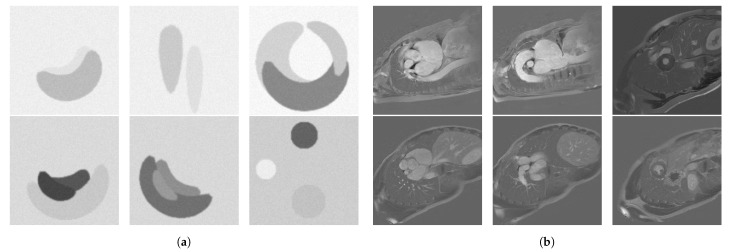
(**a**) High intensity optimization set, and (**b**) 6 representative images from the high intensity test set (imATFIB clinical study).

Upon visual assessment, we split the available MRI slices based on grey levels in two separate test sets, which we call *low intensity* and *high intensity*, respectively. To validate this split, we computed the average *signal-to-noise ratio* (SNR) over each resulted test set, and we obtained 2.570 ± 0.869 for the low intensity and 10.571 ± 3.66 for the high intensity set. The test sets contain 132 low intensity and 328 high intensity cardiac MRI slices from 32 patients and healthy volunteers from the imATFIB study. For the supervised rule optimization, we used synthetic training sets consisting of 20 low intensity and 20 high intensity images of circles of size 128 × 128 pixels. We used synthetic images because they are easy to produce, as opposed to MRI scans, and circular shapes emulate the types of structures found in our cardiac MRI. The images were filtered with a Gaussian filter and injected with Gaussian noise in order to better emulate MRI scans, and some of them were distorted. Examples from these datasets can be seen in Figure 2a,b for the high intensity set and Figure 3a,b for the low intensity set, respectively.

### 3.2. Robustness Analysis

In this section, we performed a series of preliminary experiments in order to establish a baseline for our method. For this part, we used the following optimization protocol:

1. an image from the optimization set is passed to the optimizer;
2. the rule is optimized on this image for a set number of epochs;
3. the next image is passed to the optimizer, and the global best is reset in order to avoid the particles getting stuck in local optima.

#### 3.2.1. Comparing CA−ED, $CA-ED_{pre}$ and $CA-ED_{pre-post}$

**Figure 4:**
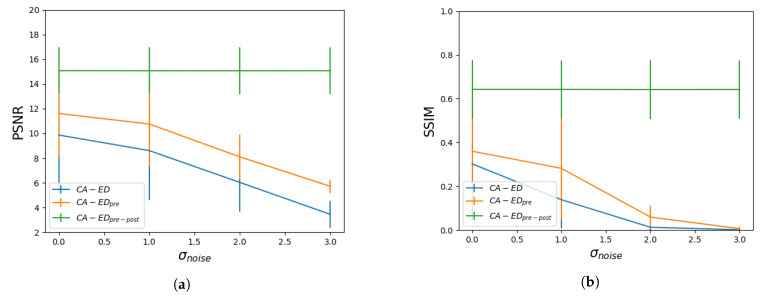

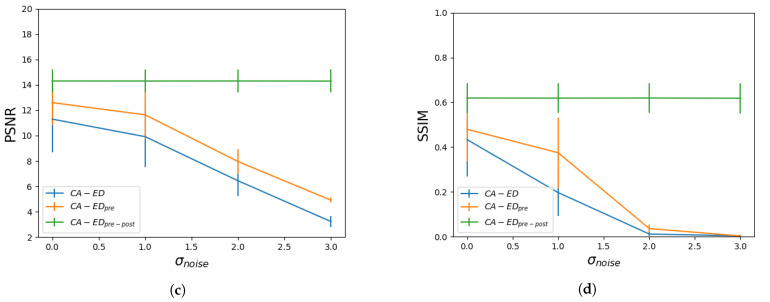
Average Peak signal-to-noise-ratio (PSNR) and Structural Similarity (SSIM) values with respect to the standard deviation of the Gaussian noise injected in the images for the low intensity dataset—(**a**,**b**), and for the high intensity dataset—(**c**,**d**).

We tested the three variations of our method under the same conditions to find the most robust one for further experiments. For this purpose, we used the original test images from each dataset, as well as the same examples injected with Gaussian noise, which we denoted by $\sigma_{noise}\in \left\{1,2,3\right\}$. We plotted the average values of the metrics with respect to $\sigma_{noise}$ for each variation in Figure 4. For $CA-ED_{pre-post}$, we pre-filtered the images with a Gaussian filter with σ=1.5.

#### 3.2.2. $CA-ED_{pre-post}$ against the Canny Edge Detector

**Figure 5:**
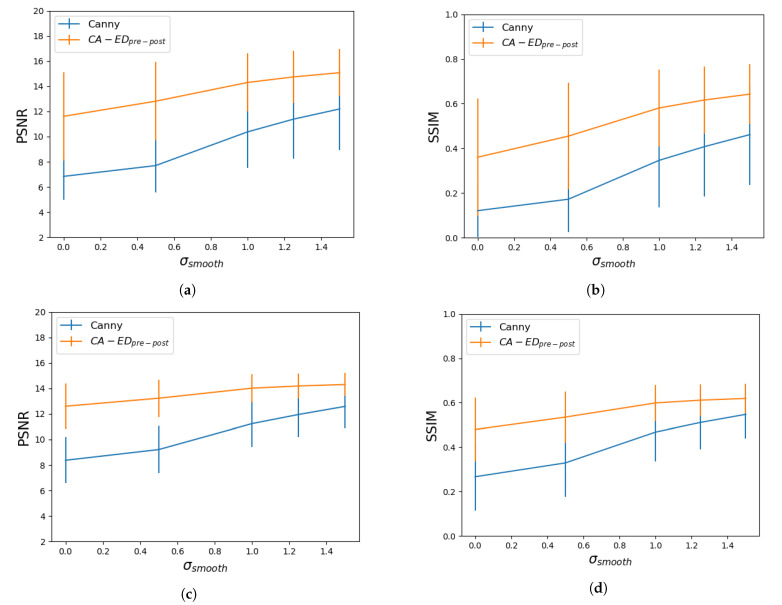
Average PSNR and SSIM values with respect to the standard deviation of the Gaussian filter used for pre-processing for the low intensity dataset—(**a**,**b**), and for the high intensity dataset—(**c**,**d**).

We further analyzed $CA-ED_{pre-post}$ by identifying the optimal value for the standard deviation of the Gaussian filter used in the pre-processing step, which we denoted by $\sigma_{smooth}$. In this regard, we averaged the metrics over each dataset obtained for several values of $\sigma_{smooth}$. In Figure 5, we show results for $\sigma_{smooth}\in \left\{0.0,0.5,1.0,1.25,1.5\right\}$.

#### 3.2.3. Optimization Analysis

In this section, we focused on evaluating the impact of using different batch sizes in the optimization of the edge detector. The batch size denotes how many images we pass to the PSO model at a time. For this part, we used the following procedure:

1. a fixed number of images from the optimization set are passed to the optimizer;
2. the rule is optimized on the batch for a set number of epochs by averaging the fitness computed for the individual images;
3. the next batch is passed to the optimizer, and the global best is reset to avoid the particles getting stuck in local optima.

#### 3.2.4. Impact of Batch Size over the Optimization Process

**Figure 6:**
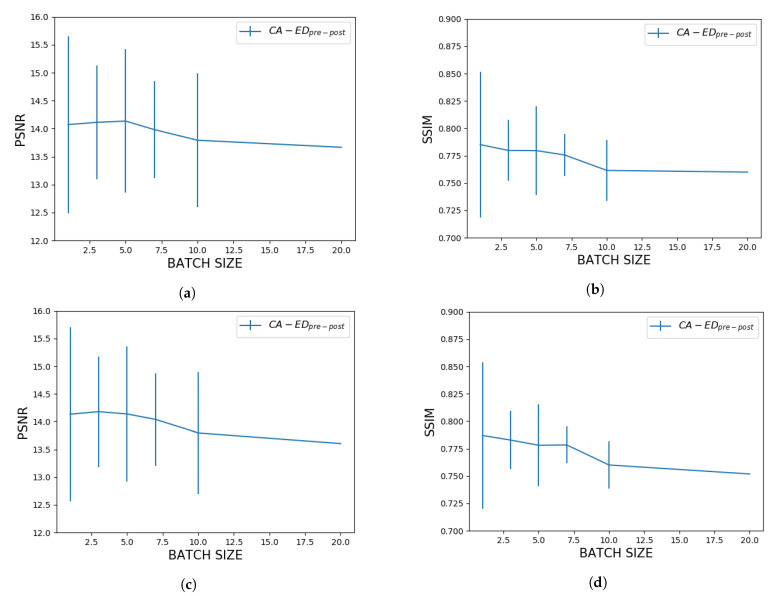
Average PSNR and SSIM values with respect to the batch size on the test images ($\sigma_{smooth}=1.5$) for the low intensity dataset—(**a**,**b**), and for the high intensity dataset—(**c**,**d**).

**Figure 7:**
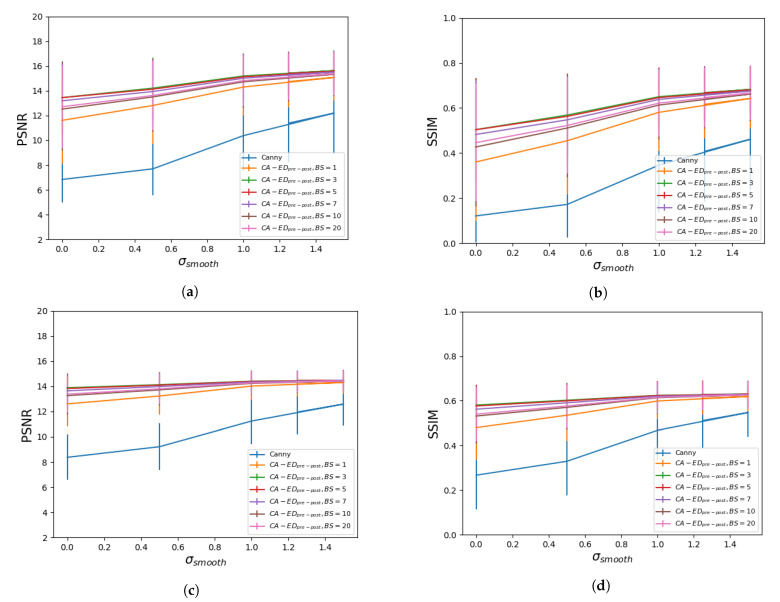
Average PSNR and SSIM values with respect to the standard deviation of the Gaussian filter used for pre-processing using different batch sizes, denoted as BS, for the low intensity dataset—(**a**,**b**), and for the high intensity dataset—(**c**,**d**).

We measured the average PSNR and SSIM values of $CA-ED_{pre-post}$ with $\sigma_{smooth}=1.5$, where the rule was optimized by passing a set number of images at a time. For our experiments we used fixed batch sizes of 1, 3, 5, 7, 10, and 20 images. In machine learning, training data is passed in batches for computational efficiency. To be able to use the same technique for a PSO model, we need to assess whether the batch size affects the result of the optimization process or not. In Figure 6, we show the average metrics with respect to the batch size for the low intensity and high intensity datasets. To provide a more general analysis, in Figure 7, we also plotted the metrics with respect to the batch size for all the values of $\sigma_{smooth}$ that we tested before. This shows that the previous results are a function of the optimization of the rule and not of the CA-based component.

#### 3.2.5. Evaluating the Difficulty of Edge Detection with Respect to Batch Size

For both datasets, we wanted to see if the differences in results can be justified by the difficulty of the edge detection problem. The low intensity dataset consists of dark, high-contrast images, while the high intensity dataset has opposite properties. From an edge detection perspective, these datasets pose very different problems.

**Figure 8:**
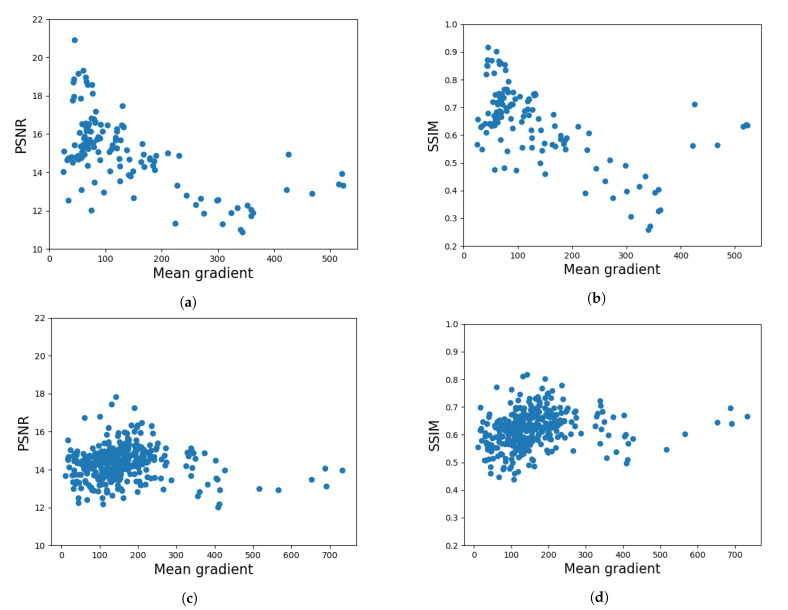
PSNR and SSIM values for each image with respect to the mean gradient ($\sigma_{smooth}=1.5$) for the low intensity dataset—(**a**,**b**), and for the high intensity dataset—(**c**,**d**).

We measured the difficulty of an image by computing the mean gradient, using the Sobel operator [30]. In Figure 8, we plotted the metrics obtained for each image in the test set with respect to the mean gradient to see how difficulty affects the metrics. In addition, we computed the Pearson correlation between the average PSNR and SSIM values and the mean gradient averaged over the entire dataset, as shown in Table 1.

## 4. Discussion

### 4.1. Robustness Analysis

#### 4.1.1. Comparing CA−ED, $CA-ED_{pre}$, and $CA-ED_{pre-post}$

We performed a preliminary experiment to determine whether adding pre- and post-processing to the CA-based edge detector improves the final result. The results in Figure 4 show a clear advantage of $CA-ED_{pre-post}$.

We see that CA−ED and $CA-ED_{pre}$ have a similar progression with respect to the noise level in the test images; however, the baseline metrics on the original images are the best for $CA-ED_{pre-post}$ and the worst for CA−ED. Moreover, for both datasets PSNR decreases monotonously because the amount of noise in the edge image increases along with the $\sigma_{noise}$. In the case of SSIM, we notice that it converges to the same value for CA−ED and $CA-ED_{pre}$ with respect to $\sigma_{noise}$ although there is a larger gap at $\sigma_{noise}=1$. This is because, for lower amounts of noise, the post-processing does help clear out incorrect (disconnected) edge points; however, higher amounts of noise produce more false positive edge points, which are more likely to be connected to one another; thus, they are not removed in the post-processing step. This affects the structural similarity with respect to the ground truth as a result. The numbers are comparable among the two datasets, and we see that $CA-ED_{pre-post}$ remains robust due to the Gaussian filtering step, which successfully clears out the noise without removing relevant edge information.

#### 4.1.2. $CA-ED_{pre-post}$ against the Canny Edge Detector

We compared the best-performing edge detector, $CA-ED_{pre-post}$, with Canny by testing several values of the $\sigma_{smooth}$ parameter. The results in Figure 5 show that $CA-ED_{pre-post}$ performs better for each value. Previous results on a different dataset [17] showed that $CA-ED_{pre-post}$ reached its optimal result for a lower value of $\sigma_{smooth}$ than Canny (an advantage since heavier pre-filtering has a higher chance of removing edge information); however, in this case, they both peak at $\sigma_{smooth}=1.5$, mainly due to the difficulty of edge detection on the cardiac MRI datasets. It is also worth mentioning that our method performs better than Canny with no pre-filtering ($\sigma_{smooth}=0$), and this gap is larger in the low intensity dataset.

### 4.2. Optimization Analysis

#### 4.2.1. Impact of Batch Size over the Optimization Process

Figure 6 shows that on our MRI datasets a batch size of 3 images is optimal for the best results. In Figure 7 we confirm this by testing $CA-ED_{pre-post}$ with multiple values for $\sigma_{smooth}$, and they are consistent with the first set of curves. In particular, for the high intensity dataset, we observe a more stable behavior with respect to variations in the level of pre-processing. Furthermore, for this dataset, the metrics converge to closer values at higher $\sigma_{smooth}$, which is also reflected in Figure 6.

#### 4.2.2. Evaluating the Difficulty of Edge Detection with Respect to Batch Size

Based on the above presented results, we analyze the different behavior of the two datasets with respect to the batch size. For this, we measure the difficulty of the test images and we plot these values for each data point with respect to the PSNR and SSIM values in Figure 8. We observe that, in the case of the low intensity dataset, the edge detector produces better edges for the images with a lower difficulty score, especially from the perspective of structural similarity. Furthermore, in Table 1, we see a negative correlation between difficulty and metrics for the low intensity images, which is absent in the case of high intensity images. Computing the median difficulty scores for the two datasets, we find a median score of 138.25 with a range of 723.62 for the low intensity dataset and a median score of 85.69 with a range of 498.58 for the high intensity dataset. Given that there is high variance in the difficulty scores, we may infer that the difference in these results is given by the SNR of the dataset, meaning that, in the case of noisier images (low intensity), the choice of batch size has a higher impact on the final results.

## 5. Conclusions

We performed an extensive comparative analysis of our CA-based edge detector with respect to Canny, a state-of-the-art method for edge detection. We see an improvement in our CA-based method after introducing the pre- and post-processing steps in terms of overall edge quality and robustness to noise. The proposed method performed better than Canny on average on our cardiac MRI dataset, on unaltered images, as well as on images with various amounts of injected noise. Additionally, we analyzed the impact of the number of images fed to the optimizer at each step on the optimization process. We found that for datasets with higher levels of noise choosing an optimal batch size can aid the optimizer in finding a suitable edge detection rule on that dataset.

As opposed to other edge detectors, our method adapts to the target images through the supervised optimization framework. In the case of cardiac MRI scans, we showed that our CA-based detector can optimize a transferable edge detection rule in a supervised manner from synthetic data, without the need of medical data annotated by experts. Our method is thus suitable for aiding the identification of cardiac structures on medical images and can be used as a component of a radiology decision support tool.

As future improvements, we are studying the extension of the CA model to the 3D space by working on volumetric images and also transferring the rules to other types of medical images and anatomical structures. Additionally, we are considering a more extensive comparison with related edge detection methods from the literature. 

## Figures and Tables

**Table 1:** Correlation between the PSNR and SSIM values and the difficulty of the test images given by the mean gradient.

	Pearson Correlation (Low Intensity Set)	Pearson Correlation (High Intensity Set)
PSNR	−0.605	−0.049
SSIM	−0.580	0.191

## References

[1] Wang R. (2016). Edge Detection Using Convolutional Neural Network.

[2] Khan A.R., Choudhury P.P., Dihidar K., Mitra S., Sarkar P. (1997). VLSI architecture of a cellular automata machine. Comput. Math. Appl..

[3] Dioșan L., Andreica A., Enescu A. (2017). The Use of Simple Cellular Automata in Image Processing. Stud. Univ. Babes-Bolyai Inform..

[4] Schiff J.L. (2011). Cellular Automata: A Discrete View of the World (Wiley Series in Discrete Mathematics & Optimization).

[5] Mohammed J., Nayak D.R. An efficient edge detection technique by two dimensional rectangular cellular automata. Proceedings of the International Conference on Information Communication and Embedded Systems (ICICES2014).

[6] Angulo K., Gil D., Espitia H. (2020). Method for Edges Detection in Digital Images through the Use of Cellular Automata.

[7] Mărginean R., Andreica A., Dioşan L., Bálint Z. (2020). Butterfly Effect in Chaotic Image Segmentation. Entropy.

[8] Amrogowicz S., Zhao Y., Zhao Y. (2016). An edge detection method using outer Totalistic Cellular Automata. Neurocomputing.

[9] Mohammad H.M., Sadeghi S., Rezvanian A., Meybodi M.R. (2015). Cellular edge detection: Combining cellular automata and cellular learning automata. Int. J. Electron. Commun..

[10] Uguz S., Sahin U., Sahin F. (2015). Edge detection with fuzzy cellular automata transition function optimized by PSO. Comput. Electr. Eng..

[11] Gonzalez C.I., Melin P., Castro J.R., Castillo O., Mendoza O. (2016). Optimization of interval type-2 fuzzy systems for image edge detection. Appl. Soft Comput..

[12] Olivas F., Valdez F., Castillo O., Melin P. (2016). Dynamic Parameter Adaptation in Particle Swarm Optimization Using Interval Type-2 Fuzzy Logic. Soft Comput..

[13] Vikhar P.A. Evolutionary algorithms: A critical review and its future prospects. Proceedings of the 2016 International Conference on Global Trends in Signal Processing, Information Computing and Communication (ICGTSPICC).

[14] Kennedy J., Eberhart R. Particle swarm optimization. Proceedings of the ICNN’95—International Conference on Neural Networks.

[15] Weikert D., Mai S., Mostaghim S. (2020). Particle Swarm Contour Search Algorithm. Entropy.

[16] Dumitru D., Andreica A., Dioşan L., Balint Z. Evolutionary Curriculum Learning Approach for Transferable Cellular Automata Rule Optimization. Proceedings of the 2020 Genetic and Evolutionary Computation Conference Companion.

[17] Dumitru D., Andreica A., Dioşan L., Bálint Z. (2020). Robustness analysis of transferable cellular automata rules optimized for edge detection. Procedia Comput. Sci..

[18] Hersey I. (1968). Textures: A Photographic Album for Artists and Designers by Phil Brodatz. Leonardo.

[19] Vilalta R., Giraud-Carrier C., Brazdil P., Soares C., Sammut C., Webb G.I. (2010). Inductive Transfer. Encyclopedia of Machine Learning.

[20] Litjens G., Ciompi F., Wolterink J.M., de Vos B.D., Leiner T., Teuwen J., Išgum I. (2019). State-of-the-Art Deep Learning in Cardiovascular Image Analysis. JACC Cardiovasc. Imaging.

[21] Jain R., Kasturi R., Schunck B.G. (1995). Edge Detection.

[22] Ziou D., Tabbone S. (1998). Edge Detection Techniques-An Overview. Pattern Recognit. Image Anal. C.

[23] Canny J. (1986). A computational approach to edge detection. IEEE Trans. Pattern Anal. Mach. Intell..

[24] Neumann J.V., Burks A.W. (1966). Theory of Self-Reproducing Automata.

[25] Kari J. (2005). Theory of Cellular Automata: A Survey. Theor. Comput. Sci..

[26] Dice L.R. (1945). Measures of the Amount of Ecologic Association Between Species. Ecology.

[27] Van der Walt S., Schönberger J.L., Nunez-Iglesias J., Boulogne F., Warner J.D., Yager N., Gouillart E., Yu T. (2014). scikit-image: Image processing in Python. PeerJ.

[28] Xess M., Agnes S.A. (2014). Analysis of Image Segmentation Methods Based on Performance Evaluation Parameters. Int. J. Comput. Eng. Res..

[29] Zhou W., Bovik A.C., Sheikh H.R., Simoncelli E.P. (2004). Image quality assessment: From error visibility to structural similarity. IEEE Trans. Image Process..

[30] Sobel I. (1968). An Isotropic 3x3 Image Gradient Operator. Present. Stanf. A.I. Proj..

